# Obesity-Altered Adipose Stem Cells Promote Radiation Resistance of Estrogen Receptor Positive Breast Cancer through Paracrine Signaling

**DOI:** 10.3390/ijms21082722

**Published:** 2020-04-15

**Authors:** Rachel A. Sabol, Vidal A. Villela, Alexandra Denys, Benjamin T. Freeman, Alifiani B. Hartono, Rachel M. Wise, Mark A. A. Harrison, Maxwell B. Sandler, Fokhrul Hossain, Lucio Miele, Bruce A. Bunnell

**Affiliations:** 1 Center for Stem Cell Research, Tulane University School of Medicine, New Orleans, LA 70112, USA; rsabol@tulane.edu (R.A.S.); vvillela@tulane.edu (V.A.V.); acote1@lsuhsc.edu (A.D.); rwise@tulane.edu (R.M.W.); mharri26@tulane.edu (M.A.A.H.); msandle1@tulane.edu (M.B.S.); 2 Department of Structural and Cellular Biology, Tulane University School of Medicine, Tulane Cancer Center, New Orleans, LA 70112, USA; bfreema1@tulane.edu; 3 Pathology and Laboratory Medicine, Tulane University School of Medicine, New Orleans, LA 70112, USA; ahartono@tulane.edu; 4 Louisiana State University Health Sciences Center (LSUHSC), Department of Genetics, New Orleans, LA 70112, USA; fhossa@lsuhsc.edu (F.H.); lmiele@lsuhsc.edu (L.M.); 5 Louisiana Cancer Research Center (LCRC), Stanley S. Scott Cancer Center, LSUSHC, New Orleans, LA 70112, USA; 6 Department of Pharmacology, Tulane University, New Orleans, LA 70112, USA; 7 Division of Regenerative Medicine, Tulane National Primate Research Center, Covington, LA 70433, USA

**Keywords:** adipose stem cells, obesity, breast cancer, radiotherapy, radiation resistance

## Abstract

Obesity is associated with poorer responses to chemo- and radiation therapy for breast cancer, which leads to higher mortality rates for obese women who develop breast cancer. Adipose stem cells (ASCs) are an integral stromal component of the tumor microenvironment (TME). In this study, the effects of obesity-altered ASCs (obASCs) on estrogen receptor positive breast cancer cell’s (ER^+^BCCs) response to radiotherapy (RT) were evaluated. We determined that BCCs had a decreased apoptotic index and increased surviving fraction following RT when co-cultured with obASCs compared to lnASCs or non-co-cultured cells. Further, obASCs reduced oxidative stress and induced IL-6 expression in co-cultured BCCs after radiation. obASCs produce increased levels of leptin relative to ASCs from normal-weight individuals (lnASCs). obASCs upregulate the expression of IL-6 compared to non-co-cultured BCCs, but BCCs co-cultured with leptin knockdown obASCs did not upregulate IL-6. The impact of shLeptin obASCs on radiation resistance of ER^+^BCCs demonstrate a decreased radioprotective ability compared to shControl obASCs. Key NOTCH signaling players were enhanced in ER^+^BBCs following co-culture with shCtrl obASCs but not shLep obASCs. This work demonstrates that obesity-altered ASCs, via enhanced secretion of leptin, promote IL-6 and NOTCH signaling pathways in ER^+^BCCs leading to radiation resistance.

## 1. Introduction

Breast cancer (BC) is the most prevalent and second most fatal cancer in women, claiming approximately 40,000 lives every year [1,2]. BC prognosis has improved over the past two decades due to better screening methods and improving therapies; however, challenges associated with standard therapy remain unresolved. Radiotherapy (RT) has been a treatment for many malignancies, including breast cancer, for over a century and has been shown to substantially increase patient survival [1]. The main challenges associated with this treatment are damage to the surrounding normal tissue and radioresistance, characterized by the unresponsiveness of some cancer cells to RT. RT is a mainstay of BC therapy. Breast-conserving therapy, which is indicated for both invasive BC and ductal carcinoma in situ, is comprised of lumpectomy and RT. An overview of BC randomized control trials demonstrated no difference in outcomes between mastectomy and breast conserving therapy [3,4]. Further, RT reduces the long-term risk for local recurrence [5]. 

Beyond RT, systemic treatments such as endocrine therapy are also a mainstay in BC treatment; however, flexible guidelines and patient preferences often leave therapy choice to physicians’ discretion although endocrine therapy is recommended for patients whose cancer display any level of estrogen expression [6,7]. Notably, it has been found that endocrine therapy and RT can be safely concurrently administered without danger of weakening disease control [6]. 

Obesity has been implicated as a risk factor in RT resistance, metastasis, and overall poor prognosis in breast cancer [8,9,10]. Furthermore, moderate to severe obesity increases the likelihood of BC recurrence and BC-specific mortality [10,11]. Obesity may promote resistance to RT through metabolic dysregulation and altered signaling axis [8]. Women who receive whole breast RT were 12.6% more likely to have regional recurrence after 5 years if they had two or more risk factors for breast cancer, including obesity [12]. Obese breast cancer patients do not respond as well to standard of care when compared to healthy weight patients. However, the mechanism(s) through which obesity alters the tumor microenvironment (TME) leading to radiation resistance have yet to be determined. It was hypothesized that obesity-altered adipose stem cells (obASCs) may be a key player in the TME player that mediates obesity driven radiation resistance. 

Adipose stem cells (ASCs) are mesenchymal lineage stem cells resident in fat tissue that are known to be recruited to sites of inflammation, including tumors [13,14]. In inflammatory environments, ASCs secrete paracrine factors such as adipokines, cytokines, and growth factors to promote regeneration and wound healing [14,15]. ASCs play an important role in the TME and can lead to more aggressive tumors. Obesity results in chronic low-grade inflammation of adipose tissue, which alters the biology of resident ASCs [16,17]. ASCs from obese individuals (obASCs, BMI > 30) secrete more proinflammatory cytokines and increased levels of leptin [15,18,19]. In previous work, Strong et al. demonstrated that obASCs enhance ER^+^BC cell proliferation through estrogen dependent pathways. Further, it has previously been shown that obASCs promote proliferation and metastasis of breast cancer compared to ASCs from healthy weight (lean) donors (lnASCs, BMI < 25) or breast cancer alone in vitro and in vivo [18,20]. obASCs promote estrogen receptor positive breast cancer (ER^+^BC) through leptin upregulation of ERα and aromatase [20]. Further, in ER^+^BC with estrogen receptor alpha mutations that lead to constitutive activation and ligand independent signaling, obASCs were shown to promote metastasis but not proliferation or tumor growth compared to lnASCs or cell grafts without ASCs [21]. Additionally, increased production of leptin from obASCs compared to lnASCs promoted triple negative breast cancer (TNBC) metastasis through upregulation of epithelial-to-mesenchymal transition genes [18,20]. In this study, we focus on the role obASCs play in response to RT of ER^+^BCCs as approximately 80% of breast cancer cases are ER+ [22]. Given these prior studies, we hypothesize that obASCs could alter ER^+^BC response to RT. Investigation of this interaction provides evidence of altered stromal cell biology within the TME that promotes RT resistance. We also demonstrate on a cellular and molecular level the signaling pathways driving increased mortality and decreased efficacy of standard treatment seen in obese BC patients.

## 2. Results

### 2.1. Obesity-Altered ASCs Promote Radiation Resistance of ER^+^BCCs Lines

**Figure 1:**
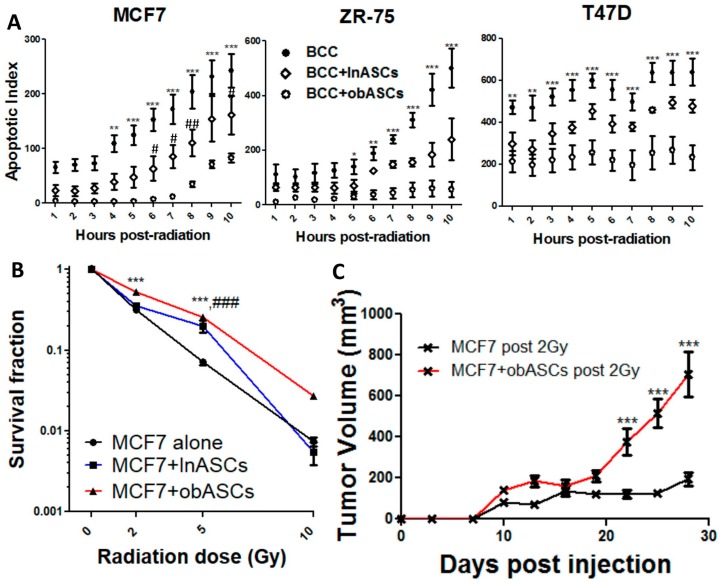
Obesity-altered adipose stem cells (obASCs) promote radioresistance of estrogen receptor positive (ER+) breast cancer. (**A**) Quantification of time-lapse imaging for 10 h after co-culture and radiation demonstrates decreased apoptotic index (CellEvent fluorescent green positive cells) of ER^+^BCC lines after radiation if co-cultured with obASCs. Breast cancer cell (BCC) lines and ASCs were co-cultured via transwell for 96 h. BCCs were irradiated at a dose of 2 Gy and then live cell time-lapse microscopy was used to quantify apoptotic cells after radiation. (**B**) Survival fraction of MCF7 after co-culture and radiation revealed increased survival fraction at 2 and 5 Gy of cells co-cultured with obASCs (* ctrl vs. obASCs; # ctrl vs. lean ASCs (lnASCs)). (**C**) In vivo tumorigenesis assay demonstrated that MCF7 co-cultured with obASCs and irradiated prior to injection had increased tumor growth compared to cells that were not co-cultured before radiation. Values reported are the mean of three independent experiments each performed in triplicate. Data from animal experiments represent an *n* = 5 animals per group with bilateral tumors. Bars, ± SEM. (* = obASCs compared to control; # lnASCs compared to control) * *p* < 0.05, ** *p* < 0.01, *** *p* < 0.001; # *p* < 0.05, ## *p* < 0.01, ### *p* < 0.001.

Time-lapse imaging of MCF7, ZR-75, and T47D cell lines alone or co-cultured with six pooled donors of lnASCs or obASCs and gamma irradiated at a dose of 2 Gy demonstrates that ionizing radiation (IR) induces apoptosis more efficiently in BCCs alone compared to BCCs that had previously been transwell co-cultured with ASCs. In all three cell lines, BCCs co-cultured with obASCs conferred a larger radioprotective effect than either BCCs alone or co-cultured with lnASCs (Figure 1A and Figure S1A). To evaluate the tumor growth of the viable cells after radiation, the surviving fraction of MCF7 was assessed at increasing doses of gamma radiation ranging from 0, 2, 5, and 10 Gy doses. The data indicate that viable BCCs after transwell co-culture with obASCs had a significantly increased surviving fraction after radiation compared to BCCs alone at both 2 and 5 Gy of radiation exposure while there was a similar yet non-statistically significant observation at 10 Gy of radiation exposure. On the other hand, BCCs co-cultured with lnASCs only had significantly increased survival at 5 Gy compared to control (Figure 1B). While there was no significant difference between lnASCs and obASCs after 5 Gy (Figure 1B), there is a difference in apoptosis between lnASCs and obASCs (Figure 1A). To evaluate the relapse potential of MCF7 co-cultured and irradiated at 2 Gy, an in vivo tumor assay was performed by injecting irradiated MCF7 cells that had been cultured in isolation or together with obASCs into ovariectomized immunocompromised mice. MCF7 cell exposed to obASCs prior to irradiation displayed significantly increased tumor growth and tumor weight at endpoint compared to MCF7 that were irradiated, but not co-cultured (Figure 1C and Figure S1C). Additionally, tumor growth and weight at endpoint of MCF7 co-cultured with obASCs prior to irradiation showed comparable tumor growth to neither non-irradiated, non-co-cultured cells nor non-irradiated, co-cultured cells (Figure 1C and Figure S1B,C).

#### obASCs Promote Radiation Resistance through Decreased Oxidative Stress in ER^+^BCCs and Promoting a Higher Percentage of Cells in S-Phase

**Figure 2:**
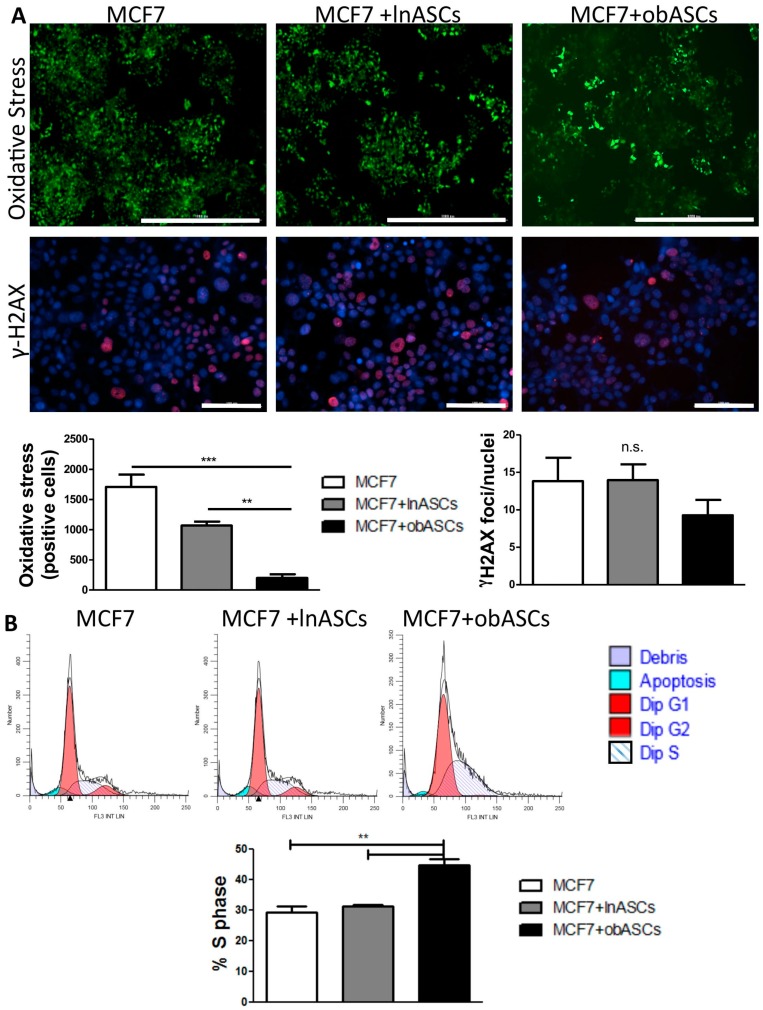
obASCs decrease oxidative stress in breast cancer cells after radiation but have no effect on DNA damage. (**A**) DCFDA, which becomes fluorescent by reacting with oxidative species, was used to quantify number of cells undergoing oxidative stress after radiation. Quantification demonstrates that there is a significant decreased in cells that were co-cultured with obASCs undergoing oxidative stress. Evaluation of double stranded DNA breaks via immunofluorescent γ-H2AX staining with Hoechst 33342 nuclear counterstain revealed no difference in DNA damage after radiation between groups. Scale bar represents 1000 μm in the upper panel and 100 μm in the lower panel. (**B**) Cell cycle analysis using propidium iodide (PI) staining and ModFit software demonstrates percent of cells in the different phases of the cell cycle. Quantification reveals increased percent of diploid cells in S-phase after transwell co-culture with obASCs. Values reported are the mean of three independent experiments each performed in triplicate. Bars, ± SEM. ** *p* < 0.01, *** *p* < 0.001.

To asses potential mechanisms of survival following IR, oxidative stress in BCCs was evaluated after co-culture at 2 Gy RT and found that co-culture with obASCs significantly reduced DCFDA oxidation in ER^+^BCCs (201.3 ± 61.0 positive cells (mean ± SEM)) compared to lnASCs (1068.0 ± 66.9) or non-co-cultured cells (1707 ± 204.3) (Figure 2A). Next the levels of DNA damage were evaluated, as this is a consequence of oxidative stress, using γ-H2AX foci/nuclei as a marker of double stranded breaks after radiation. No significant difference in double stranded DNA damage was found after co-culture and radiation across groups (Figure 2A). To evaluate additional mechanisms through which obASCs may be promoting radioresistance, we evaluated percentage of BCCs in the various phases of the cell cycle after co-culture with lnASCs or obASCs because S-phase is associated with radiation resistance [23,24,25]. We cultured cells in serum free media for 48 h to synchronize cells. Then BCCs were cultured in either complete culture media (CCM) for 24 h or a transwell insert containing lnASCs or obASCs in CCM that was introduced to BCCs for 24 h. Cell cycle analysis was performed using propidium iodide and flow cytometry and we found that cells cultured with obASCs had significantly increased percentage of cells in S-phase (44.72 ± 2.16; mean ± SEM) compared to BCCs + lnASCs (31.37 ± 0.30) and BCCs + CCM (29.34 ± 1.89) (Figure 2B). These data demonstrate that obASCs have a radioprotective effect by increasing the percent of cells in S-phase and reducing oxidative stress in BCCs after radiation, but did not affect DNA damage in BCCs after radiation.

### 2.2. obASCs Produce Increased Leptin, which Promotes Radiation Resistance through IL-6 Upregulation

**Figure 3:**
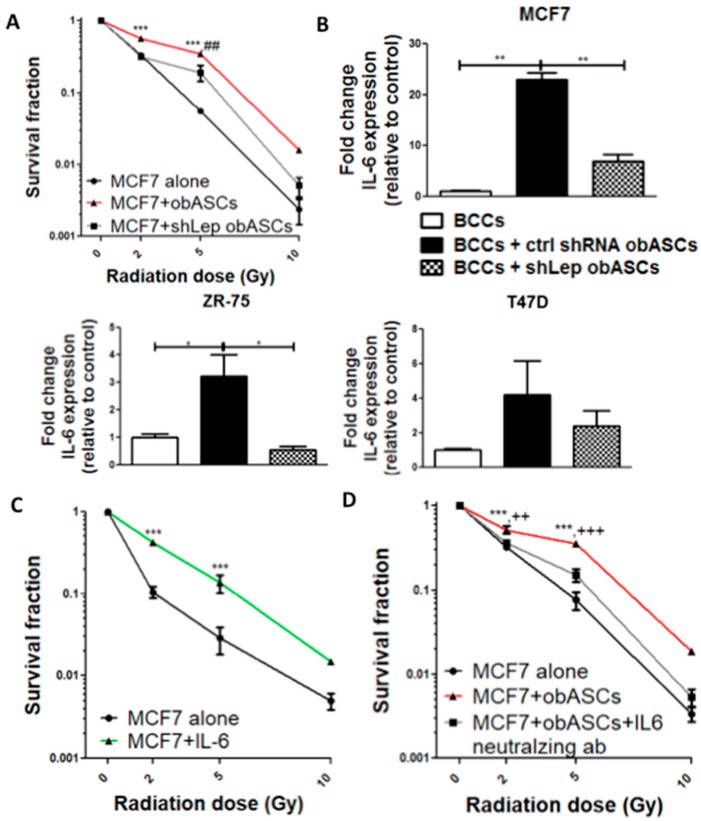
Leptin from obASCs confers radiation resistance through upregulation of IL-6. (**A**) Stable knockdown of leptin in obASCs through shRNA abrogated the pro-survival effect of obASCs on breast cancer (BC) after radiation (* ctrl vs. shCtrl obASCs; # ctrl vs. shLep obASCs). (**B**) shCtrl obASCs upregulated IL-6 expression in ER^+^BCCs while shCtrl lnASCs and shLep obASCs did not upregulate IL-6. (**C**) Cells treated with 20 ng/mL of recombinant human IL-6 for 3 days and irradiated have increased radiation resistance. (**D**) IL-6 neutralizing antibody added to co-culture with obASCs also abrogated the pro-survival effect of obASCs (* ctrl vs. obASCs; **+** obASCs vs. obASCs with IL-6 neutralizing antibody). Values reported are the mean of three independent experiments each performed in triplicate. Bars, ± SEM. * *p* < 0.05, ** *p* < 0.01, *** *p* < 0.001, ++ *p* < 0.01 and +++ *p* < 0.001.

Our team has previously shown that obASCs produce increased levels of leptin compared with lnASCs [20]. To determine if leptin from obASCs promoted survival following IR, BCCs were co-cultured with obASCs that had stable knockdown of leptin via shRNA (shLep) or obASCs with control shRNA (shCtrl) constructs. The surviving fraction of MCF7 BCCs cultured alone, with shCtrl obASCs, or with shLep obASCs demonstrates that shCtrl obASCs confer a significant survival benefit over non co-cultured cells at 2 and 5 Gy. MCF7s co-cultured with shLep obASCs had decreased surviving fraction compared to shCtrl obASCs, but still conferred some survival benefit over control after RT. Thus, while leptin from obASCs is playing a large role, other secreted factors from obASCs are likely also promoting survival benefit (Figure 3A).

To evaluate a potential mechanism to explain the partial survival benefit of shLep obASCs over shCtrl ASCs, the expression of both IL-6 and IL-1α, which have been shown to promote stemness and radiation resistance, were assessed by RT-qPCR [26,27]. IL-1α expression was undetectable in control and co-cultured MCF7, T47D, and ZR-75 cell lines (40 cycles). However, a significant difference in expression of IL-6 was identified in ER^+^BCCs from three different cell lines (MCF7, ZR-75, and T47D). ER^+^BCCs were cultured alone or co-cultured with shCtrl lnASCs, shCtrl obASCs, or shLep obASCs. Across all three cell lines, BCCs co-cultured with shCtrl obASCs demonstrated an upregulation of IL-6 expression compared to non-co-cultured control cells, BCCs cultured with shCtrl lnASCs, and BCCs cultured with shLep obASCs (Figure 3B). The surviving fraction of MCF7s treated with a physiologic dose (20 ng/mL) of recombinant IL-6 prior to radiation demonstrated similar survival benefit to obASCs and had a significant increase in surviving fraction at 2 and 5 Gy compared to untreated MCF7 (Figure 3C). Further, addition of an IL-6 neutralizing antibody to the co-cultures abrogated the survival benefit of BCCs co-cultured with obASCs. obASCs conferred significant survival compared to both control and obASCs + IL-6 neutralizing antibody at a dose of both 2 and 5 Gy and trended to have survival benefit at 10 Gy (Figure 3D).

### 2.3. Leptin from obASCs Promotes a Cancer Stem-Like Phenotype through NOTCH Signaling

**Figure 4:**
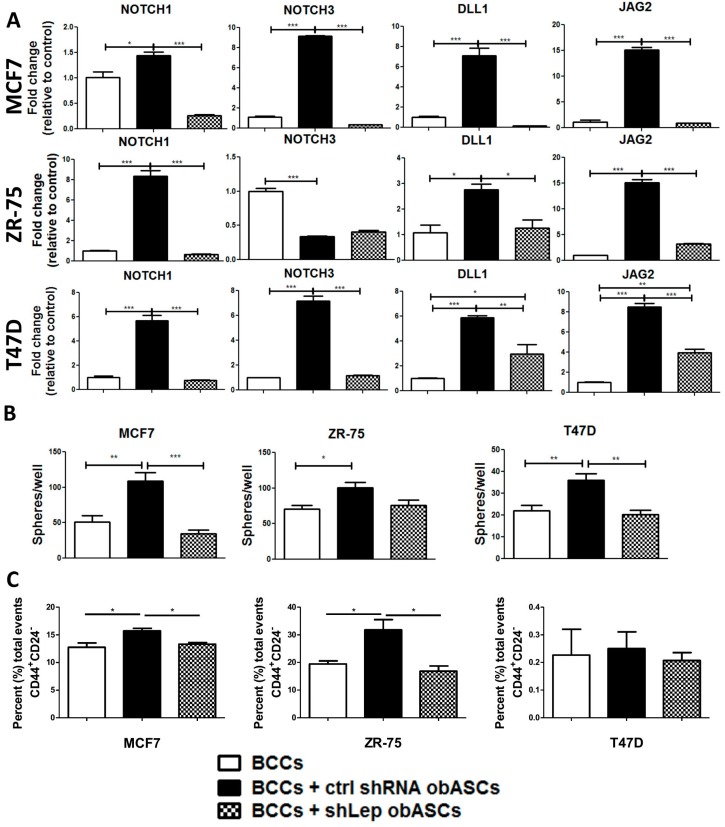
obASCs promote a cancer stem-like phenotype through leptin. (**A**) Transwell co-culture with shCtrl obASCs upregulates NOTCH1, NOTCH3, DELTA1, and JAGGED2 in ER^+^BCCs. (**B**) BCCs have increased ability to form mammospheres after co-culture with shCtrl obASCs followed by 2 Gy radiation compared to non-co-cultured cells or cells co-cultured with shLep obASCs. (**C**) Flow cytometric analysis of the breast cancer stem cell markers CD44^+^CD24^−^ was evaluated for BCCs after transwell co-culture with shCtrl obASCs or shLep obASCs. Values reported are the mean of three independent experiments each performed in triplicate. Bars, ± SEM. * *p* < 0.05, ** *p* < 0.01, *** *p* < 0.001.

BCCs from three separate cell lines (MCF7, ZR-75, and T47D) were cultured alone, with shCtrl obASCs, or with shLep obASCs and analyzed for changes in mRNA expression of genes associated with the NOTCH signaling pathway. Genes screened include DLL1, JAG1, JAG 2, NOTCH1, NOTCH2, NOTCH3, NOTCH4, DLL3, DLL4, CDKN1A, and WNT4. BCCs co-cultured with shCtrl obASCs had significant upregulation of NOTCH signaling pathway members; however, BCCs co-cultured with shLep obASCs did not demonstrate this enrichment for NOTCH associated genes. Specifically, all three ER^+^BCCs showed significant upregulation of NOTCH1 (1.45, 5.68, 8.34-fold) (MCF7, T47D, ZR-75), DLL1 (7.08, 5.89, 2.76-fold) (MCF7, T47D, ZR-75), JAG1 (2.28, 13.42, 8.63-fold) (MCF7, T47D, ZR-75), and JAG2 (15.11, 8.49, 15.16-fold) (MCF7, T47D, ZR-75) as well as significant upregulation of other NOTCH players that are cell line dependent, demonstrating an overall enrichment of NOTCH gene expression by shCtrl obASCs that is not upregulated by shLep obASCs (Figure 4 and Figure S2). NOTCH signaling is associated with a cancer stem-like phenotype, therapeutic resistance, and increased mortality [28,29]. 

Next, we evaluated if leptin from obASCs altered primary mammosphere formation of BCCs after co-culture and 2 Gy IR. Here we evaluated primary spheres to evaluate in a 3D system post-radiation survival of BCCs that were primed with shCtrl obASCs or shLep obASCs. We found that shCtrl obASCs significantly increased sphere forming ability of MCF7, T47D, and ZR-75 following 2 Gy IR. However, BCCs co-cultured with shLep obASCs before RT showed no significant difference in sphere forming ability compared to non-co-cultured BCCs (Figure 4B). Additionally, we evaluated the breast cancer stem cell phenotype (CD44^+^CD24^−^) using flow cytometry and found a higher percent of total events of the phenotype CD44^+^CD24^−^ in BCCs co-cultured with shCtrl obASCs compared to BCCs co-cultured with shLep obASCs or control BCCs in both MCF7 and ZR-75, while T47D showed no difference in the CD44^+^CD24^−^ cell phenotype between groups and overall had almost undetectable levels of this cancer stem cell phenotype through flow cytometry (Figure 4C).

## 3. Discussion

In this study, we demonstrate that obASCs upregulate IL-6 and NOTCH expression in BCCs in part through leptin, which promotes RT resistance of ER^+^BC. While leptin produced from obASCs is the focus of this study, these data may translate more broadly as it is well known that obese individuals have significantly higher levels of circulating leptin [30]. This preclinical study is important because it demonstrates that normal tissue surrounding a tumor can affect tumor biology and ultimately ascribe therapeutic resistance. These data provide cellular evidence of leptin-IL6-NOTCH signaling from obASCs promoting radiation resistance. This provides a possible mechanism contributing to the poor response of obese women to standard therapies resulting in increased morbidity and mortality. More studies in this field are warranted; however, these data suggest development and implementation of precision therapies targeting this pathway in obese patients receiving RT for breast cancer. 

Notch has been shown to promote self renewal and cell proliferation [31]. Lagadec et al. have demonstrated that several NOTCH receptor-ligand parings are upregulated in BCCs following radiation exposure in a dose-dependent pattern [31]. In non-small cell lung cancers, tumors with high NOTCH activity are more proliferative and radioresistant than tumors with normal NOTCH activity [32,33]. Furthermore, patients whose tumors show high NOTCH activity have worse prognoses than those with otherwise normal NOTCH activity [32]. Increased NOTCH signaling also plays a significant role in chemoresistant pancreatic cancer, where downregulation of this same pathway has been shown to reduce the cancer’s invasiveness and partially reverse the EMT phenotype [34]. In addition to poor prognosis and association with metastasis, NOTCH is essential for angiogenesis and growth of breast cancers, a pathway in which Leptin also plays a role [35].

Leptin is an adipokine found in higher levels in ASCs isolated from obese individuals as compared to lean individuals [36]. Leptin-induced NOTCH signaling contributes to BC proliferation, metastasis, and correlates to BC development in the context of obesity [36]. In breast and pancreatic cancer cells, leptin upregulates NOTCH receptors, ligands, and targets [36,37]. Leptin has pro-tumorigenic effects, such as increasing cancer cell proliferation, self-renewal, angiogenesis, and survival [36,37]. Leptin has also been shown to induce resistance to both Fulvestrant and Tamoxifen in ER^+^BCCs [36].

The broad acting inflammatory cytokine IL-6 has been demonstrated to play a key role in numerous cancers [26,38,39]. High levels of IL-6 have been implicated in promoting epithelial–mesenchymal transition and stem cell-like properties in cancer cells [38]. Elevated IL-6 levels in BC patients’ tumors and serum are associated with a poor prognosis [26,40]. In addition, IL-6 promotes BC bone metastasis via the NOTCH signaling pathway [41]. IL-6 induces STAT3 activation, which, in turn, promotes proliferation, inhibits apoptosis, and contributes to radiation resistance [39]. Research shows that hormone resistant prostate cancer cells have increased IL-6 expression and activated STAT3 [39]. IL-6 overexpression is also positively linked to radiation resistance [39]. A study by Wu et al. demonstrated that IL-6 inhibition of radioresistant prostate cancer sensitized the cancer to radiotherapy [39]. Inhibition of STAT3 has also been shown to reverse the radioresistant phenotype in breast cancer cells [42].

## 4. Materials and Methods 

### 4.1. Adipose Stem Cells

All protocols were reviewed and approved by the Pennington Biomedical Research Center Institutional Review Board (PBRC #23040 approved in December 2011) (LaCell, New Orleans, LA, USA). All methods were performed in accordance with relevant institutional guidelines and regulations. Subjects provided informed written consent (PBRC #23040). ASCs were isolated from normally discarded subcutaneous abdominal adipose tissue from 12 Caucasian females (two groups, six donors/group) undergoing elective liposuction procedures, as previously described [18,20]. ASCs were isolated from lipoaspirate of subcutaneous adipose tissue isolated from obese women (BMI > 30) or lean women (BMI < 25). Lipoaspirate was washed with phosphate buffered saline (PBS), incubated at 37 °C in a rocking incubator at 100 rpm for 1 h in 0.1% collagenase type 1 (Sigma, St. Louis, MO, USA) and 1% powdered bovine serum albumin (Sigma) dissolved in 1 mL/g tissue in PBS. Digested tissue was then centrifuged to remove lipids, primary adipocytes, and collagenase solution leaving behind the stromal vascular fraction in the cell pellet. Cells were suspended in complete culture media (CCM), which consisted of alpha-minimal essential media (αMEM; Gibco; Grand Island, NY, USA), 10% fetal bovine serum (Atlanta Biologicals, Lawrenceville GA, USA), 100 units per mL penicillin/100ug/mL streptomycin (P/S; Gibco), and 2 mM L-Glutamine (Gibco) and plated on T175 culture flasks. Media was replaced every 3–4 days until the cells achieved 70% confluence. At 70% confluence cells were harvested with 0.25% trypsin/1mMEDTA (Gibco) and cryopreserved in liquid nitrogen. The average BMI for the donor groups is lnASCs (22.7 ± 1.9; *n* = 6) obASCs (32.7 ± 3.7; *n* = 6). 

For all experiments, sub-confluent cells (<70% confluent) between passages 2 and 6 were used. ASCs were characterized as previously described [18]. Stable transfection of ASCs with a construct targeting leptin and a construct targeting a non-human gene as a negative control was performed as previously described [20]. 

### 4.2. Cell Culture

Breast cancer cells MCF7, T47D, and ZR-75 cells were purchased from American Type Culture Collection (ATCC, Manassas, VA, USA). Cells were cultured in estrogen deprived complete culture media (CCM) made up of phenol free α-MEM (Gibco, Grand Island, NY, USA) with 10% heat inactivated charcoal dextran stripped FBS (Atlanta Biologicals, Flowery Branch, GA, USA), with 1% L-glutamine (Gibco), and 1% anti-anti (100×) antibiotic-antimycotic (Gibco). Cells were grown at 37 °C in 5% humidified CO_2_ and media was changed every 2–3 days. Cells were passaged when plates reached 70%–80% confluence.

ASCs were cultured on 150 cm^2^ dishes (Nunc, Rochester, NY, USA) in DMEM-F12 (Gibco), 10% lot tested heat inactivated FBS (Hyclone Laboratories, Inc., Logan, UT, USA), 1% L-glutamine (Gibco), and 1% anti-anti (Gibco). Cells were incubated at 37 °C with 5% humidified CO_2_. Media was replaced every 2–3 days. Cells were passaged when plates reached 70% confluence. shLeptin ASCs were made as previously described [20]. For all co-culture experiments obASCs and BCCs were cultured in estrogen depleted phenol free media. Where indicated, cancer cells were treated with 20 ng/mL humanized recombinant IL-6 (ThermoFisher, Waltham, MA, USA), 20 ng/mL of humanized recombinant IL-1α (BioLegend, San Diego, CA, USA) or 0.1 µg/mL IL-6 neutralizing antibody (R&D Systems, Minneapolis, MN, USA).

### 4.3. Co-Culture Studies

First, 5 × 10^4^ BCCs were plated in the bottom of a 6-well plate (Nunc) and six pooled donors of obASCs (BMI > 30) or lnASCs (BMI < 25) were seeded at a density of 5 × 10^4^ cells in a 0.4 µm pore transwell (Corning Inc., Corning, NY, USA). Cells were allowed to adhere for 24 h in their respective wells. After 24 h, transwell inserts containing ASCs were transferred to wells harboring BCCs cells for 96 h. ER^+^BCCs were then irradiated at (2, 5, 10 Gray (Gy)) using a Nordion Gammacell40 Cesium gamma irradiatior (0.692 Gy/min). BCCs with ASCs were then co-cultured for an additional 24 h. BCCs were then collected for analysis. 

### 4.4. Cell Cycle Analysis

MCF7 were seeded at a density of 5 × 10^4^ in a 6-well plate (Nunc) and six pooled donors of 5 × 10^4^ obASCs (BMI > 30) or lnASCs (BMI < 25) were seeded in a 0.4 µm pore Transwell (Corning) and allowed to adhere overnight. BCCs were serum starved for 24 h. Media was replaced with CCM and Transwells containing ASCs were introduced to BCCs for 24 h. BCCs were then harvested and cell suspensions were pelleted and washed twice with PBS. Beckman Coulter Cell Cycle Kit was used to measure quantity of DNA on a Gallios Flow Cytometer (Beckman Coulter, Brea, CA, USA) and modeled on ModFit LT Software (Verity Software House, Topsham, ME, USA). 

### 4.5. Live Cell Imaging

Cytation5 (BioTek, Winooski, VT, USA) was used for live cell imaging. Cells were stained with DCFDA (Sigma-Aldrich, St. Louis, MO, USA) for oxidative stress, CellEvent Caspase 3/7 green detection reagent for apoptotic index, and purified anti-phosphorylated human γ-H2A.X (Ser139) (BioLegend) with Hoechst 33342 (ThermoFisher) as a counterstain for double stranded DNA breaks. Cell counting was performed within the Cytation5 Software (BioTek). 

### 4.6. Surviving Fraction

Viable BCCs after radiation were counted using trypan blue (Gibco). Clonogenic assay was set up and cultured in CCM for 14 days at which time the plates were washed with PBS (Sigma) and fixed and stained with 3% crystal violet in methanol (Sigma-Aldrich) for 30 min at room temperature on a rocker. Excess stain was removed with DI water and colonies were counted manually (minimum size 200 cells/colony). Surviving fraction was adjusted for plating efficiency. Surviving fraction was calculated as described by Munshi et al. [43]. 

### 4.7. Xenograft Model

Ovariectomized SCID/beige (CB17.Cg-Prkdc^scid^Lyst^bg−1^/Crl) female mice (4–6-week-old) were obtained from Charles River Laboratory (Wilmington, MA, USA). All protocols involving animals were conducted in compliance with State and Federal law and approved by Tulane University Institutional Animal Care and Use Committee (IACUC). Animals were anesthetized with isoflurane gas and oxygen delivered by nose cone for cell graft. Mice were divided into four groups, with five animals per group: unirradiated MCF7, unirradiated MCF7 that had been exposed to obASCs for 96 h via transwell co-culture, MCF7 after 2 Gy of radiation, MCF7 that had been exposed to obASCs for 96 h via transwell co-culture after 2 Gy of radiation. Cells (1 × 10^6^ per injection) were suspended in 50 µL of PBS and 100 µL phenol free growth factor reduced Matrigel (BD Biosciences, MA, USA) and injected bilaterally into the fifth mammary fat pads. 17β Estradiol 60 day slow release pellets (Innovative Research America, Sarasota, FL, USA) were implanted in between the scapula of all animals. Tumor size was measured every 3 days using digital calipers and calculated as previously described [20]. 

### 4.8. RT-qPCR analysis

BCCs were collected and RNA was isolated with Qiazol reagent (Qiagen, Valencia, CA, USA), and purified with RNeasy columns (Qiagen) followed by treatment with DNase 1 (Qiagen). Then, 1 µg of RNA was collected from each sample for further cDNA synthesis using VILO cDNA synthesis kit (Invitrogen, Carlsbad, CA, USA). The cDNA then underwent RT-qPCR with EXPRESS SYBR Green qPCR synthesis kit (Invitrogen). RT-qPCR was performed using Bio-Rad CFX96 C1000 Thermocycler (Bio-Rad, Hercules, CA, USA), fold change (2^−(ΔΔCT)^) values were calculated, and RT-qPCR results were graphed in GraphPad Prism (La Jolla, CA, USA).

### 4.9. Mammospheres

After co-culture and a 2 Gy dose of gamma radiation BCCs were lifted, counted with trypan blue and 100,000 viable cells per well were plated in ultra-low attachment surface cell culture plates (Corning) in mammosphere media (DMEM/F-12 (Gibco) + 10% B27 (Gibco) + 10 µg/mL EGF and FGF (Invitrogen) added every 3 days). Spheres per well were counted after 7 days. 

### 4.10. Flow Cytometry

BCCs were cocultured in transwells as described above for 3 days. BCCs were harvested with trypsin and washed with PBS. Cells were then blocked with 1% BSA and 1% CD16/CD32 in PBS and stained with antibodies against CD24 (Cat #: 17-0247-41) (EBioscience) and CD44 (Cat #: 61-0441-82) (EBioscience). Samples were analyzed with a Gallios Flow Cytometer (Beckman Coulter, Brea, CA, USA) with Kaluza software (Beckman Coulter). A minimum of 5000 events were captured and analyzed.

### 4.11. Statistical Analysis

All values are presented as means ± standard error of the mean (SEM). Analysis of variance (ANOVA) with Tukey post-hoc analysis was used to determine statistical differences among three or more groups. Two-way ANOVA with Bonferroni post hoc analysis was used to determine differences for experiments with multiple groups and time points. Statistical significance was set at *p* < 0.05. Data was analyzed using GraphPad Prism software (GraphPad). 

## 5. Conclusions

Obesity-altered ASCs in the TME promote breast cancer cells to have a stem cell-like phenotype. Stem cell characteristics result in increased proliferation, metastasis, and greater resistance to conventional therapeutic measures such as chemotherapy and radiotherapy. One well-studied pathway implicated in the stem cell phenotype is the NOTCH signaling pathway. This study demonstrates that exposure to obASCs confers significant radiotherapy resistance to BCCs via pathways interacting with leptin. Leptin upregulates the NOTCH pathway expression and IL-6 in BCCs resulting in a radiation resistant BC. These data show a link between upregulation of IL-6 and NOTCH signaling pathway intermediates and ER^+^BCC survival after IR. Obesity is a risk factor for breast cancer and for increased mortality due to worse patient responses to standard therapies. Here evidence of a cellular intermediate and paracrine-signaling pathway that may contribute to worse outcomes for obese breast cancer patients is provided, one that could be targeted with precision medicine to improve prognoses for the obese patient population. 

## Supplementary Materials

The following are available online at https://www.mdpi.com/1422-0067/21/8/2722/s1.

## Abbreviations

ASCs	Adipose stem cells
TME	Tumor microenvironment
obASCs	Obesity-altered ASCs
ER^+^BCC	Estrogen receptor positive breast cancer cells
RT	Radiotherapy
lnASCs	Lean ASCs
ER^+^	Estrogen receptor positive
BC	Breast cancer
BCC	Breast cancer cells
TNBC	Triple negative breast cancer
Gy	Gray
SEM	Standard error of the mean
CCM	Complete culture media
shLep	Leptin shRNA
shCtrl	Control shRNA
BMI	Body mass index
