# Supplementary material for: Obesity-Altered Adipose Stem Cells Promote Radiation Resistance of Estrogen Receptor Positive Breast Cancer through Paracrine Signaling
Source: Int J Mol Sci. 2020 Apr 15;21(8):2722. doi: 10.3390/ijms21082722 (PMC7216284; doi:10.3390/ijms21082722)
Supplement: Supplementary file 1 [file ijms-21-02722-s001.pdf]

Obesity-altered adipose stem cells promote radiation resistance of estrogen receptor positive breast cancer through paracrine signaling

Running Title: obese ASCs promote breast cancer radioresistance

Rachel A. Sabol[1], Vidal A. Villela[1], Alexandra Denys[1], Benjamin T. Freeman[2], Alifiani B. Hartono[3], Rachel M. Wise[1], Mark A. A. Harrison[1], Maxwell B. Sandler[1], Fokhrul Hossain[4,5], Lucio Miele[4,5], Bruce A. Bunnell*[1,6,7]

[1]Center for Stem Cell Research, Tulane University School of Medicine, New Orleans LA
[2] Department of Structural and Cellular Biology, Tulane University School of Medicine, Tulane Cancer Center, New Orleans, LA
[3] Pathology and laboratory Medicine, Tulane University School of Medicine, New Orleans LA
[4]Louisiana State University Health Sciences Center (LSUHSC), Department of Genetics, New Orleans, LA
[5]Louisiana Cancer Research Center (LCRC), Stanley S. Scott Cancer Center, LSUSHC, New Orleans, LA
[6]Department of Pharmacology, Tulane University, New Orleans LA
[7]Division of Regenerative Medicine, Tulane National Primate Research Center, Covington, LA

Corresponding Author: Bruce A. Bunnell, PhD, 1430 Tulane Ave, #8699 New Orleans

LA, 70112; (504) 988-3329; bbunnell@tulane.edu

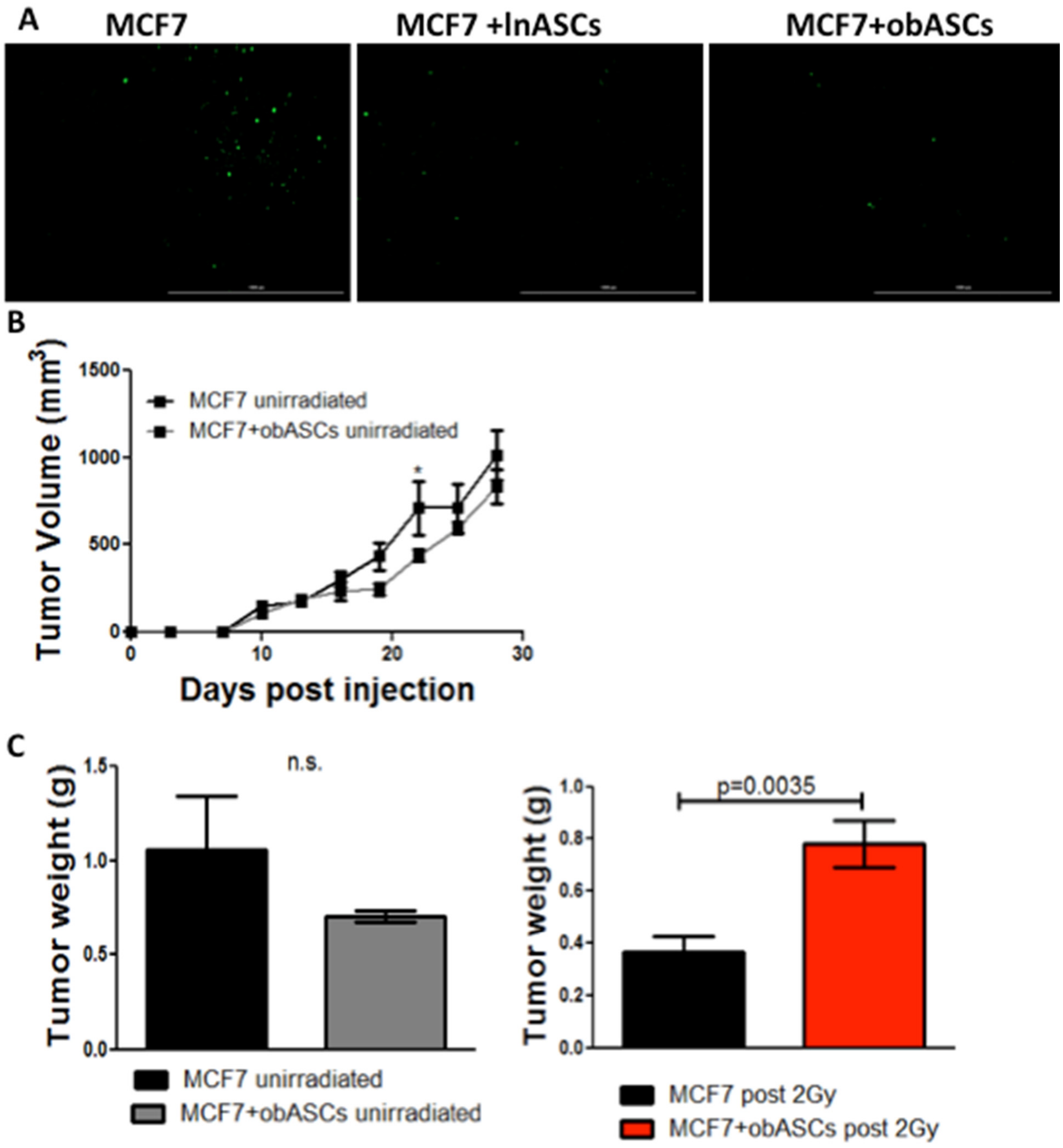

Supplemental Figure 1: obASCs promote radiation resistance, but do not affect tumor growth of non-irradiated cells. A) Representative videos of time-lapse imaging promotes decreased apoptosis in MCF7 after co-culture with obASCs prior to radiation (dose 2Gy). B) Unirradiated cells injected into the mammary fat pads reveals that MCF7 co-cultured with obASCs prior to injection did not affect tumor growth of MCF7 xenografts. C) Tumor weight at endpoint from irradiated and unirradiated groups are shown. Data from animal experiments represent an n=5 animals per group with bilateral tumors. Bars represent mean ± SEM.

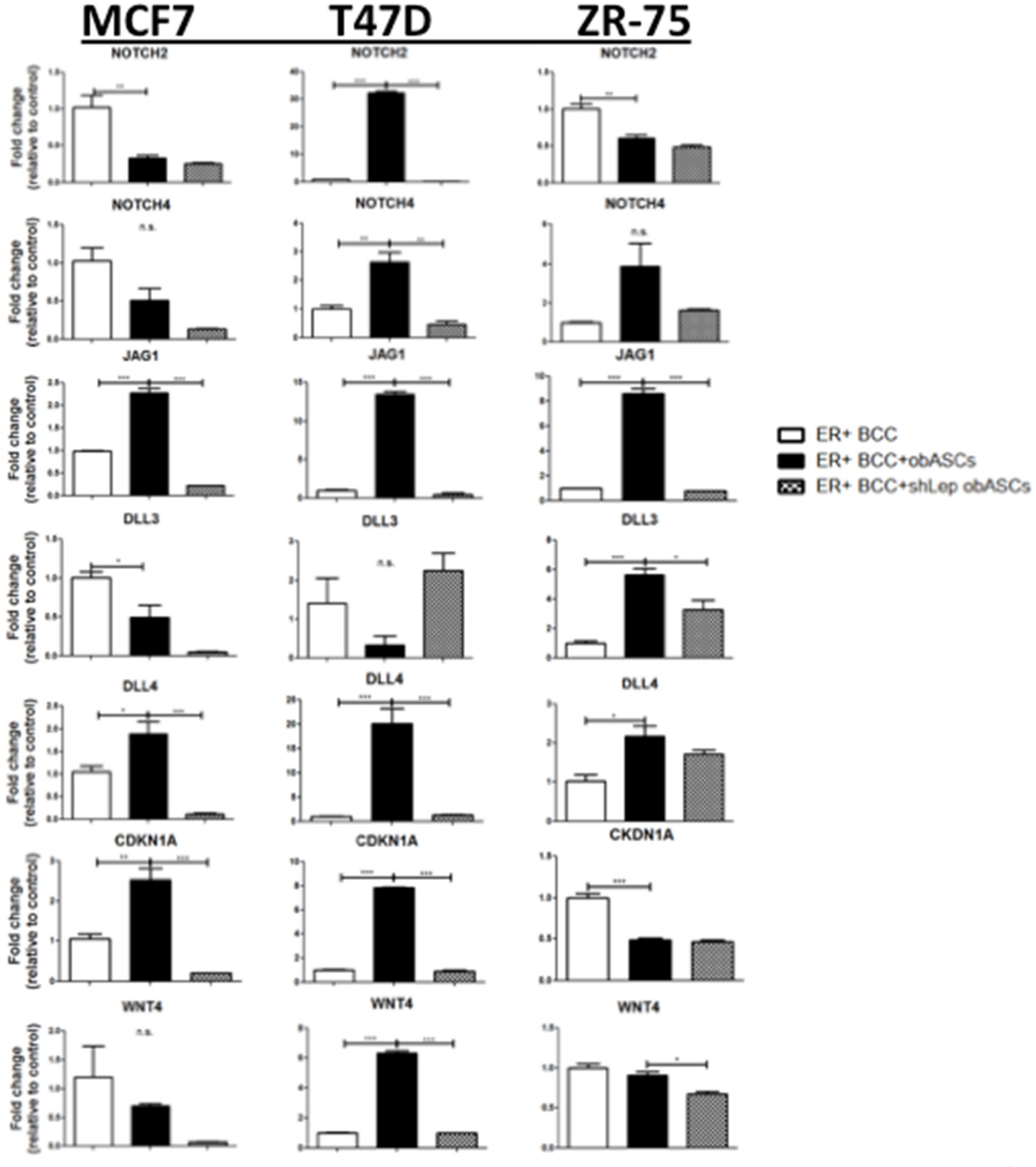

Supplemental Figure 2: Leptin produced by obASCs upregulates gene expression of NOTCH signaling components in ER⁺ BC. Co-culture with obASCs up regulated NOTCH signaling pathway players; however, co-culture with shLep obASCs does not up regulate NOTCH signaling.. Values reported are the mean of three independent experiments each performed in triplicate. Bars represent mean ± SEM. (*=obASCs compared to control) *$p<0.05$, ** $p<0.01$, ***$p<0.001$.

Original western blot uncropped: Membrane was cut before probing with primary antibody for NOTCH1 or Actin. Wells 1-3 correspond to ZR75 alone, ZR75 + shCtrl obASCs, and ZR 75+shLep obASCs in supp fig 2B respectively and wells 7-9 correspond to T47D alone, T47d+shctrl obASCs, and t47D+shLep obASCs respectively.

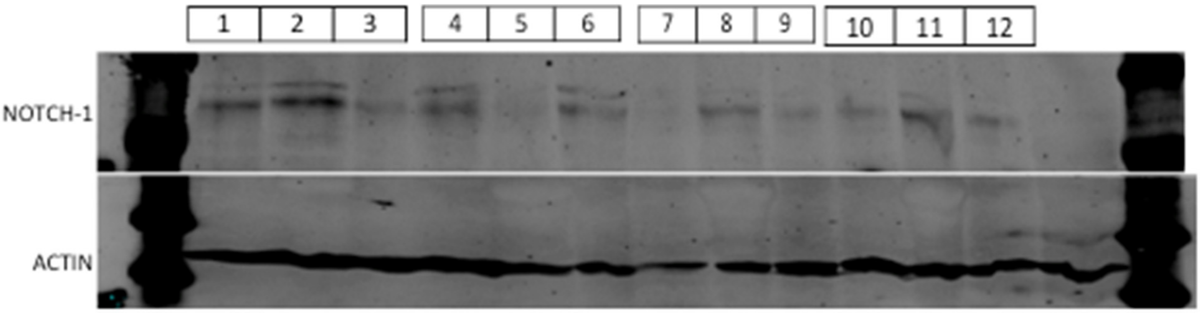
1
2
3
4
5
6
7
8
9
10
11
12
NOTCH-1
ACTIN
